# *Leishmania* ITS1 Is Genetically Divergent in Asymptomatic and Symptomatic Visceral Leishmaniasis: Results of a Study in Southern Iran

**DOI:** 10.1155/2020/5351098

**Published:** 2020-08-28

**Authors:** Zahra Rezaei, Eqlima Azarang, Saeed Shahabi, Mostafa Omidian, Bahman Pourabbas, Bahador Sarkari

**Affiliations:** ^1^Professor Alborzi Clinical Microbiology Research Center, Shiraz University of Medical Sciences, Shiraz, Iran; ^2^Department of Parasitology and Mycology, School of Medicine, Shiraz University of Medical Sciences, Shiraz, Iran; ^3^Basic Sciences in Infectious Diseases Research Center, Shiraz University of Medical Sciences, Shiraz, Iran

## Abstract

It has been documented that the genotypic traits in symptomatic and asymptomatic cases of visceral leishmaniasis (VL) may be different. The current study aimed to find out and compare the genotype and intraspecies diversity of *Leishmania* Internal Transcribed Spacer 1 (ITS1) from asymptomatic and symptomatic VL cases in southern Iran. *Methods*. Buffy coat samples from seven VL patients, with clinical signs and symptoms, and seven asymptomatic VL cases, were evaluated in this study. Samples of asymptomatic individuals were obtained from children living in a VL endemic area in southern Iran, while the samples of symptomatic subjects were obtained from patients admitted to hospitals with a diagnosis of VL. DNA was extracted from the buffy coats of the samples and PCR-amplified, targeting the ITS1of *Leishmania*. The PCR products were sequenced, and the consensus sequences were assembled and multiple-aligned with a set of *Leishmania* strains retrieved from the GenBank, using Clustal W. The phylogenetic tree was rooted, using MEGAX software, and the diversities based on haplotype and nucleotides, as well as the number of polymorphic sites, were measured using DnaSP v5.0 software. The results of ITS1 sequencing in 5 out of 7 asymptomatic VL cases showed 99.25% to 100% similarity with the *Leishmania infantum* ITS1 sequence (accessed number: MN648746), and one isolate was considered as just *Leishmania* sp. In one sample, 99.75% similarity was seen with the ITS1 sequence of *Crithidia fasciculata*. Of the symptomatic VL patients, the PCR product revealed a 340 bp band corresponding to *L. infantum* in all of the samples. By analyzing the ITS1 sequences, all seven sequences formed a clade somewhat different from other *Leishmania* species and considered as *Leishmania* sp. Haplotype and nucleotide diversity were much more prevalent in symptomatic cases where six haplotypes were seen in the ITS1 of *Leishmania* from symptomatic patients and only two haplotypes were observed in the samples from asymptomatic cases. The findings of the current study showed that the *Leishmania* ITS1 from symptomatic VL and asymptomatic cases has significant genetic differences. Besides, infection with *Crithidia fasciculata* was reported, for the first time, in an asymptomatic case, which deserves further study.

## 1. Introduction

Leishmaniasis continues to be one of the health challenges in many countries of the world, with an estimated annual worldwide incidence of 700,000 to 1,300,000 for cutaneous leishmaniasis (CL) and approximately 500,000 cases for visceral leishmaniasis (VL) [1, 2]. The disease is caused by different species of *Leishmania* parasites and can produce a range of clinical symptoms based on the type of parasite involved [3, 4]. The spectrum begins in a general category of localized or diffuse skin involvement, which is a self-limiting skin condition. Also, the parasite can cause skin-mucosal involvement and eventually cause systemic and fatal disease by involving the viscera and internal organs.

In VL, most cases do not progress to overt disease and remain asymptomatic [5–7]. The proportion of asymptomatic to symptomatic VL cases has been reported to be 4 : 1 in Kenya, 11:1 in Sudan, and 9 : 1 in India and Nepal [8–10]. In asymptomatic VL cases, there are parasites in their peripheral blood circulation that can be detected by molecular methods or even culture [11–13]. Asymptomatic humans or animals may constitute a reservoir for VL and contribute a risk of distributing the VL to another human or animals [5, 13–16].

Although most of the seroconverted asymptomatic VL cases do not develop an acute form of the disease, however, it has been shown that the progression to the overt form of the disease is associated with high positive qPCR, as well as rK39 ELISA or DAT antibody titers [17]. Accordingly, it has been suggested that healthy people living in given VL endemic areas having high antibody titers or being qPCR positive have an increased chance to progress to VL disease [17]. qPCR on blood samples showed 500 times less parasitemia in asymptomatic cases in comparison with the symptomatic ones in India [12].

Several studies documented that the genotypic traits in symptomatic and asymptomatic cases of VL may vary [18, 19]. A study in southern France showed a significant genetic difference between *Leishmania* species, isolated from asymptomatic and symptomatic patients, especially those with HIV/VL coinfection [18]. The study also revealed that the isolates from asymptomatic cases have a weak polymorphism in their parasitic genome [18].

Apart from the parasite genotype, the host's genetic background can be involved in creating asymptomatic or symptomatic forms of VL [20]. It has been shown that the symptomatic VL is associated with the gene encoding a receptor for transforming growth factor beta (TGF-*β*) whereas the asymptomatic trait is linked to the gene encoding IIa receptor for the Fc fragment of IgG [20]. However, the association between SNP/HLA genotyping and progression from asymptomatic or seroconversion to VL overt disease has been insignificant [17]. Yet, another study in Brazil reported an association between the VL phenotype with SNP on chromosome 9 and 19 [21].

The current study aimed to find out and compare the genotype and intraspecies diversity of *Leishmania* isolated from asymptomatic and symptomatic VL cases in southern Iran.

## 2. Materials and Methods

### 2.1. Study Subjects

The protocol of this study was approved by the Ethical Committee of Shiraz University of Medical Sciences (SUMS). Informed consent was obtained from the patients or their guardians in the case of children. The subjects of this study were two groups of people. The first group consisted of 7 children admitted to Nemazee Hospital, Shiraz University of Medical Sciences, Shiraz, Iran, with clinical signs and symptoms of the disease. These VL patients had more than two weeks of fever, had hepatosplenomegaly, and they were all positive in IFAT (titer > 64), using an Iranian strain of *L. infantum* (MCAN/IR/14/M14) as an antigen and confirmed by a real-time PCR and rK39-rapid diagnostic test (Kalazar Detect™ Rapid Test for VL, InBios, Seattle, USA). They all recovered after either antimonial or amphotericin B therapy. The second group consisted of seven asymptomatic children who lived in the Sarmahshed area of Kazeroun County, one of the main endemic foci of VL in the south of Iran [13, 22, 23]. The children included 4 girls and 3 boys, ranging in age from 1 to 12 years. These children were selected from those who had been confirmed for *Leishmania* infection in our previous study, by using the ELISA system using crude antigen of Iranian strain of *L. infantum* (MCAN/IR/14/M14) and molecular methods [13]. The presence of *Leishmania* infection in these children was confirmed by PCR, using ITS2 primers. These children had no clinical signs or symptoms of VL, had no previous history of the disease, and have not developed the overt disease after one year of follow-up. Also, there were not any active VL cases in the children's family members during sample collection.

### 2.2. DNA Extraction and Nested-PCR

For both groups of the subjects, DNA was extracted from the buffy coats of whole blood (5 ml) of each subject, using a commercial kit. PCR was performed on the extracted DNA to amplify the ITS1 fragment, using LITSR (ITS1-F) CTGGATCATTTTCCGATG and L5-8S-(ITS1-R) TGATACCACTTATCGCACTT primers [24, 25]. The PCR program was set as follows: initial denaturation at 95°C for 5 min, followed by 30 cycles at 94°C for 30 s, 55°C for 45 s, and 72°C for 1 min and a final extension at 72°C for 5 min in an Eppendorf thermocycler. PCR products were separated by electrophoresis on 1.5% agarose gel. The PCR products were sequenced bilaterally for the ITS1 genomic fragment, using the same pair of primers used in the PCR assay.

### 2.3. Phylogenetic and Genetic Analysis

Chromas program, as implemented in the software BioEdit version 7.2.5, was used to view and analyze the nucleotide sequences [26]. Consensus sequences were assembled and multiple-aligned with a set of *Leishmania* strains retrieved from the GenBank, using Clustal W implemented in the above mentioned software [26]. The final aligned sequences with a total of 344 positions were converted in the FASTA and MEGA format for further analyse by using MEGAX software [27]. Phylogenetic relationships were reconstructed, using a Bayesian inference (BI) tree in MRBAYES, version 3.1.2 [28]. The Bayesian inference was performed with two simultaneous runs and four search chains within each run (three heated chains and one cold chain) for 10,000,000 generations, sampling trees every 1000 generations using the Markov chain Monte Carlo method. In our analysis, we left the prior on-state frequencies at its default setting (prset statefreqpr = Dirichlet (1, 1, 1, 1)). The structure of the substitution model was determined by setting of lset nst = 6 and rates = equal (flat Dirichlet (1, 1, 1, 1, 1, 1) distribution). The default appropriate setting of the prior for the proportion of invariable sites was set with a uniform distribution between 0 and 1. The prior for shape of scaled gamma distribution of site rates and also for the proportion of invariable sites was considered as uniform. For topology, the prior was considered as an equal probability on all distinct, and for a partition-specific rate multiplier, it was fixed (1.0).

The reliability of nodes was assessed using the Bayesian posterior probability for Bayesian analysis. Trees were rooted with the sequence of *Crithidia fasciculata* (Accession no. MT302171). Genetic diversity based on haplotype diversity (Hd) and nucleotide diversity (*π*), as well as the number of polymorphic sites, were measured, using DnaSP v5.0 software.

## 3. Results

**Figure 1:**
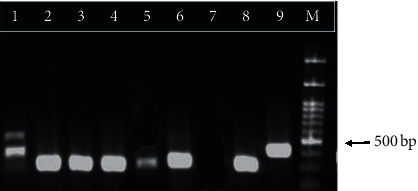
PCR product electrophoresis for ITS1 fragments of *Leishmania* from asymptomatic children on 1.5% agarose gel. Lane 1–6: isolated samples from asymptomatic children; lane 7: negative control; lane 8: *L. infantum* positive control; lane 9: *L. tropica* positive control; and lane M: 1 kb molecular weight marker.

Electrophoresis of the PCR product of ITS1 fragments of *Leishmania* isolates from asymptomatic subjects revealed a 340 bp band in six samples corresponding to *L. infantum* (Figure 1). DNA of *L. infantum* and *L. tropica* were used as the positive controls. ITS1 sequencing results for the 7 asymptomatic cases showed that 5 of the samples had 100% similarity to the ITS1 sequence of *L. infantum*, with an accession no. MN648746, registered in the GenBank (lanes 1, 3, 4, 5 and 6 in Figure 1). One of the samples was similar to the ITS1 sequence of the *Leishmania* genome (lane 7 in Figure 1). The most surprising one was the sequencing result of one of the samples (lane 2 in Figure 1, about 410 bp) which had 99.75% similarity with the ITS1 sequence of the parasite *Crithidia fasciculata* (accession no. HM004585).

**Figure 2:**
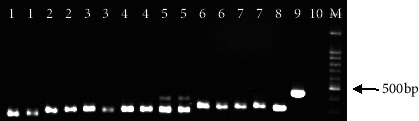
PCR product electrophoresis for ITS1 fragments of *Leishmania* from VL patients on 1.5% agarose gel. Lane 1–7 (in duplicate): *Leishmania* isolated from VL patients; 8: *L. infantum* positive control; 9: *L. tropica* positive control; 10: negative control; and M: 1 kb molecular weight marker.

Of the VL patients, electrophoresis of the PCR product revealed a 340 bp band corresponding to *L. infantum* in all of the samples (Figure 2).

### 3.1. Phylogenetic Analysis

**Figure 3:**
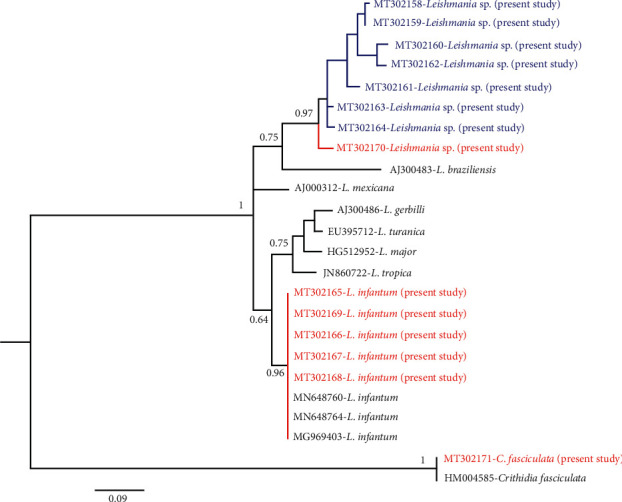
Phylogenetic tree of representative sequences of *Leishmania* from asymptomatic and symptomatic VL cases and reference sequences, based on the ITS1 fragment. Posterior probability values from the Bayesian analysis for nodes are indicated below or above branches. Samples isolated from asymptomatic and symptomatic cases are shown in red and blue color, respectively.

The nucleotide sequences generated in this study have been deposited in GenBank under accession no. MT302158-71. All seven *Leishmania* sequences isolated from symptomatic patients along with one *Leishmania* sequence isolated from an asymptomatic patient formed a clade somewhat different from other *Leishmania* species and considered as *Leishmania* sp. (Figure 3). Monophylogeny of these two clades was supported with high Bayesian posterior probability (Figure 1). A sequence of *Crithidia* (accession no. MT302171), isolated from an asymptomatic patient, was closely related with a sequence of *Crithidia fasciculata* with accession no. HM004585 (Figure 3).

### 3.2. Genetic Diversity

**Figure 4:**
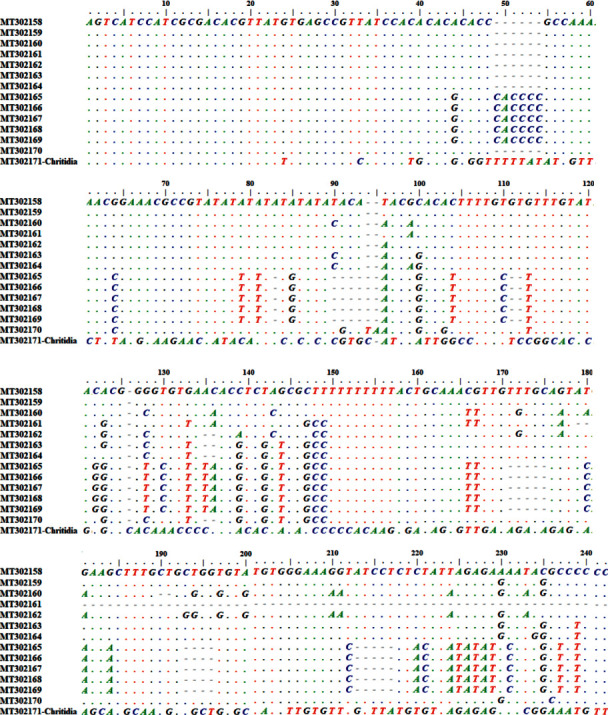
Alignments of ITS1 sequences of *Leishmania* from asymptomatic and symptomatic VL cases.

Based on the sequences of the ITS1 fragment of the isolates, 29 sites were polymorphic and 28 sites were parsimony informative, resulting in the identification of 8 haplotypes. From 28 parsimony informative sites, 27 sites had two variants and one had three variants. The number of parsimony informative sites with four variants was 3. Of the 8 observed haplotypes, 2 haplotypes were seen in the *Leishmania* sequences isolated from asymptomatic patients and six haplotypes were seen in *Leishmania* sequences isolated from symptomatic patients. No haplotype was shared between these two groups. Haplotype diversity (Hd) and nucleotide diversity (*π*) values were 0.952 and 0.059 in *Leishmania* sequences isolated from symptomatic patients, respectively, while these two diversity values (Hd and *π*) were 0.33 and 0.047 for *Leishmania* sequences isolated from asymptomatic patients. Haplotype and nucleotide diversity in the ITS1 fragment of *Leishmania* were much more prevalent in symptomatic in comparison with asymptomatic cases. The alignment of the ITS1 sequences in the current study is shown in Figure 4.

## 4. Discussion

For many years, there has been speculation that, in VL, the *Leishmania* genotypic differences involve in asymptomatic or symptomatic forms of the disease. In the present study, we used the ITS1 fragment to analyze the genetic diversity of *Leishmania* isolates in people infected with *Leishmania* but without clinical signs or symptoms and those symptomatic VL patients.

A comparison of *Leishmania* ITS1 sequences from asymptomatic cases revealed that the sequences are mainly the same with low polymorphism. This is maybe because these genotypes are derived from the same clone of the parasite circulating in the areas that have not had the opportunity to differentiate and find differences. It may also be due to the pressure of the host's immune system, which prevents the parasite from differentiating and keeps it in a nonpathogenic state [18]. The findings of the current study are consistent with those of a previous study in France which reported a very weak genetic diversity in *Leishmania* isolates from asymptomatic cases in comparison with isolates from the VL patients [18]. In the present study, six haplotypes were seen in symptomatic cases whereas only two haplotypes were observed in the ITS1 of *Leishmania* from asymptomatic cases. This finding again is consistent with the findings of Hide et al. in France who found fewer haplotypes in asymptomatic isolates in comparison with the isolates from VL disease [18].

In one of the asymptomatic cases, infection with *C. fasciculata* was detected. Since 1980, coinfections of *Leishmania* with apparently single-host trypanosomatids have been reported, some of which in individuals with healthy immune systems [29, 30]. Recently, in a study by Ghabakhloo et al., in Fars Province, where the current study was carried out, *C. fasciculata* was detected in 1.8% of CL patients [31]. The interesting point in their study was that, in patients' lesion, only *C. fasciculata* has been identified and *Leishmania* parasites have not been found. Therefore, the coinfection of *Leishmania* and *C. fasciculata* has not been the case, and CL lesion has been attributed to *C. fasciculate* [31]. Also, a recent study in Brazil reported *Crithidia*-related parasites in two fatal VL-like diseases, both of which have been resistant to current disease treatments [32]. The importance of our finding is that the presence of *Crithidia* in people living in endemic areas of VL may cause a cross reaction in serological tests and lead to false-positive results. Previous studies have confirmed the cross reaction of *Crithidia* with *Leishmania* in serological tests such as ELISA and IFA [33]. Besides, as a result of this cross reaction, VL-related seroepidemiological studies may show a higher prevalence in such areas. Although *Crithidia* or *Crithidia*-like protozoa have been reported in patients with CL or VL, the role of this protozoan in causing disease in humans has so far been in a state of ambiguity and requires extensive studies in this field.

In the current study, variation was seen in *Leishmania* ITS1 sequences in VL patients, so that even in two patients, the same sequences were not seen, and all had significant differences with each other. Previous studies have also reported similar diversity for *Leishmania* isolated from VL patients in comparison with asymptomatic cases [18, 34]. It should not be forgotten that all of the asymptomatic cases in this study were from the same area, whereas the VL patients were from different areas of Fars Province in the south of Iran. It is, therefore, recommended that asymptomatic VL cases from other areas be evaluated to see whether such homogeny exists between the isolates from different geographical regions. The main limitations of the study were the low numbers of both VL and asymptomatic cases; therefore, studying a larger population will provide robust conclusions. Another limitation of the study was the presence of VL patients from different endemic areas, while the asymptomatic individuals were all from the same area.

## 5. Conclusions

Findings of the current study showed that *Leishmania* isolated from symptomatic and asymptomatic VL cases has significant genetic diversity in the ITS1 fragment. The study also revealed a weak polymorphism in the ITS1 of *Leishmania* from asymptomatic cases, in comparison with those isolated from VL patients. Besides, infection with *Crithidia fasciculata* was reported, for the first time, in an asymptomatic VL case, which deserves further study.

## Data Availability

The data used to support the findings of this study are included within the article.
